# Loculated pericardial hematoma compressing the right atrium post mechanical aortic valve replacement and the role of point-of-care echocardiography: a case report

**DOI:** 10.1186/s13256-023-03988-w

**Published:** 2023-06-25

**Authors:** Mira Hamdan, Fady Khoury, Antoine Kossaify

**Affiliations:** Cardiology Division, Saint Esprit Kaslik University USEK, Hospital Notre Dame des Secours, PO Box # 3, Saint Charbel Street, 12345 Byblos, Lebanon

**Keywords:** Echocardiography, Cardiac tamponade, Point-of-care ultrasound (POCUS), Hematoma removal, Case report

## Abstract

**Background:**

Bleeding in the postoperative period after cardiac surgery is relatively frequent, especially in patients with early anticoagulant therapy, as in the case of mechanical valve replacement. Diffuse hemopericardium is relatively easy to diagnose; however, loculated pericardial hematomas leading to hemodynamic collapse are relatively rare and their diagnosis is more challenging.

**Case presentation:**

This report is of a 75-year-old Asian woman who presented dyspnea, confusion and hemodynamic collapse related to loculated pericardial hematoma compressing the right atrium 3 days after mechanical aortic valve replacement. Urgent transthoracic echocardiogram performed as point-of-care approach showed right atrial compression, the aortic valve prosthesis had normal function. Surgical removal of the hematoma resulted in complete recovery.

**Conclusion:**

Loculated pericardial hematoma might lead to hemodynamic collapse. Close monitoring of hemodynamic parameters is essential, also point-of-care echocardiography is essential for early recognition and prompt management in patients with critical hemodynamic condition.

## Introduction

Cardiac surgery is a relatively safe procedure when tailored patient preparation is made preoperatively, along with careful follow-up in the immediate postoperative phase [1]. However, bleeding in the postoperative period after cardiac surgery is still relatively frequent, especially in patients for whom early anticoagulant therapy is required like in the case of mechanical valve replacement [2]. Diffuse hemopericardium is relatively easy to diagnose; however, loculated pericardial hematomas leading to hemodynamic collapse are relatively rare and their diagnosis more challenging. This report is of a 75-year-old Asian woman with loculated pericardial hematoma compressing the right atrium, 3 days after mechanical aortic valve replacement, and identified by point-of-care ultrasonography (POCUS). In case of postoperative hemodynamic collapse, early detection and prompt management are mandatory for saving patients and bringing better outcome.

## Case presentation

A 75-year-old Asian woman with severe aortic stenosis was admitted for surgical aortic valve replacement, she had been experiencing exertional chest pain and dyspnea for more than 6 months. The patient’s medical history includes mild chronic obstructive pulmonary disease, type II diabetes and hypothyroidism; also she has not had any prior cardiothoracic surgery. Preoperative transthoracic echocardiography showed severe aortic valve stenosis (classical type with normal flow, high gradient), mean transaortic gradient of 55 mmHg, calcified aortic cusps with tri-leaflet morphology, diastolic dysfunction grade 1, normal left ventricular systolic function, and moderate left ventricular hypertrophy. Coronary angiogram showed normal coronaries.

After discussing the proposed procedure, along with alternative options, the decision was made to replace the aortic valve with a mechanical prosthesis, in agreement with the patient. The patient underwent mechanical aortic valve replacement without any complications. Anticoagulant therapy was initiated 12 h postoperatively, after ensuring no active bleeding. Anticoagulant therapy consisted of Enoxaparin 40 mg twice a day along with Acenocoumarol 4 mg (half tablet/day). During the first 48 h postoperatively, the patient was asymptomatic, with stable hemodynamic condition. On day three postoperatively, the international normalized ratio (INR) was 2.2, and the patient started to manifest hemodynamic dysfunction with hypotension (average blood pressure 70/40 mmHg, taken via intra-arterial probe) and oliguria. Physical examination showed tachycardia (average 120–130 bpm) with normal heart sounds intensity, jugular venous distension, and tachypnea with normal breathing sounds. The patient started deteriorating rapidly, developed hemodynamic instability and respiratory failure requiring immediate intubation and mechanical ventilation along with volume expansion, vasopressors and inotropic support.

**Fig. 1:**
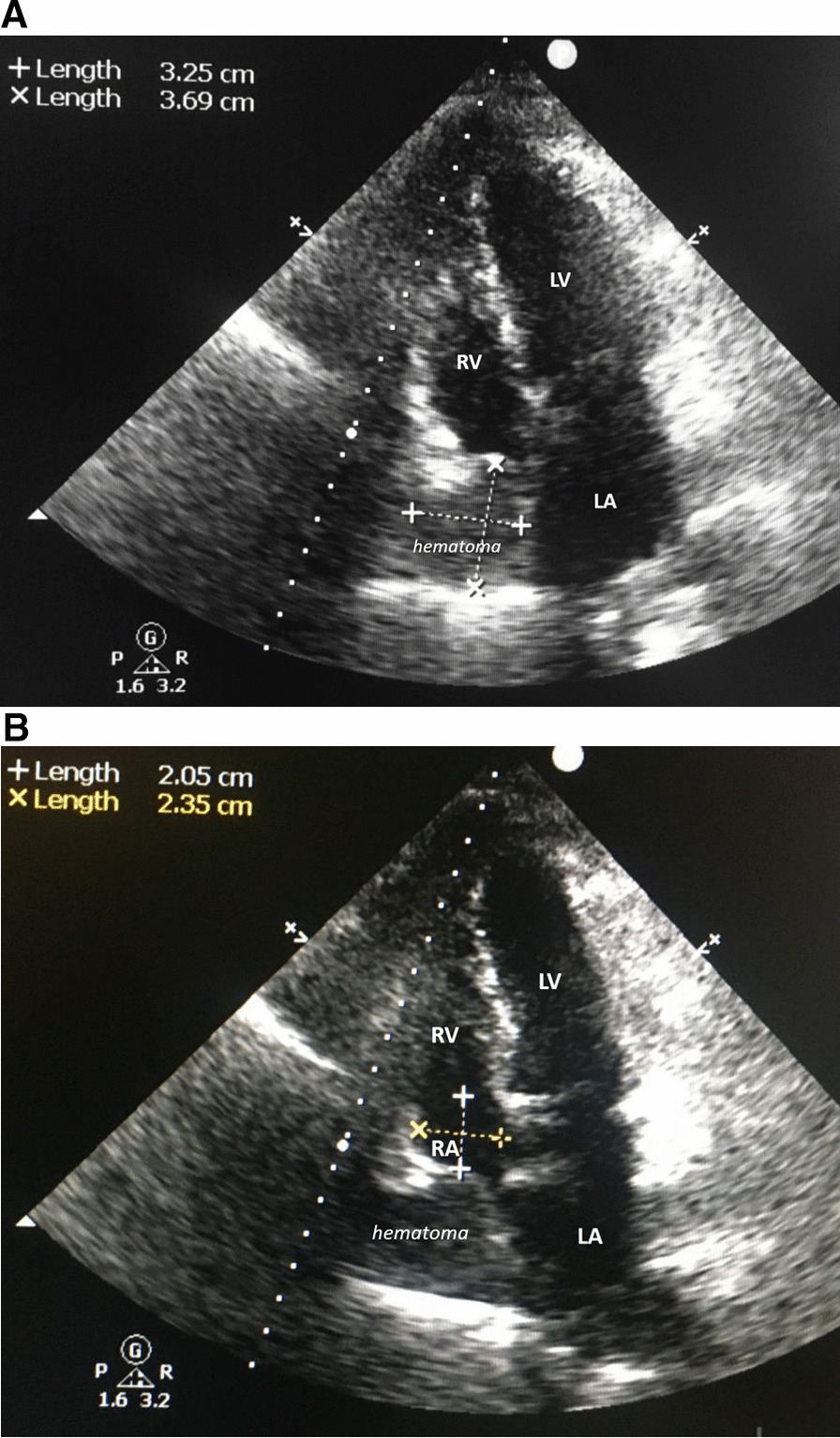
**A** Transthoracic echocardiography with apical four-chamber view, showing a round shaped 32 × 37 mm hematoma compressing the right atrium (systolic view). *LV* left ventricle, *RV* right ventricle, *LA* left atrium. **B** Transthoracic echocardiography with apical four-chamber view (after minor probe tilt) showing right atrial dimensions (20 × 23 mm) with compression by adjacent hematoma (systolic view). *LV* left ventricle, *RV* right ventricle, *LA* left atrium, *RA* right atrium (compressed)

Urgent transthoracic echocardiogram (*CX50 xMATRIX ultrasound system, Philips Medical Systems, Andover, MA, USA*) adopting mainly a qualitative approach and implemented as POCUS exam with semi-portable echo machine showed normal pericardium, no pericardial echo free space, normal function of aortic prosthesis, preserved left ventricular systolic function (as per visual estimate) with right atrial compression by a localized mass identified as probable hematoma of 32 × 37 mm; the right atrial dimensions were reduced to 20 × 23 mm (Fig. 1A, B). The right ventricle was mildly undersized compared to the preoperative dimensions, no septal bounce was documented. The patient was on respiratory machine and assessment of the inferior vena cava dimensions and compliance was difficult; similarly, evaluation of respiratory variations of the Doppler flow velocities were judged unreliable.

The patient was taken rapidly to the operative room, there was a loculated pericardial hematoma compressing the latero-anterior wall of the right atrium and prompt evacuation of the hematoma was performed, there was no active bleeding site identified. The volume of the hematoma (a mixture of coagulum and clots) was roughly estimated to be around 50 to 60 cc. Thereafter, the patient became asymptomatic, restored normal hemodynamics with normal sinus rhythm and was discharged few days later.

## Discussion

Loculated pericardial hematoma occurring after cardiac surgery might lead to hemodynamic collapse. This article presents a case of hemodynamic collapse early after aortic valve replacement, and shows the valuable advantage of teamwork and prompt intervention. Cases of hematoma compressing the atria post cardiac surgery have been reported almost 30 years ago, also hematomas with right atrial compression were reported more frequently than hematomas compressing the left atrium [3, 4]. Loculated pericardial hematoma compressing the atria might be difficult to diagnose and might lead to severe hemodynamic collapse [5].

Bleeding and hematoma are more common in case of early anticoagulant therapy as it was the case in this patient who required anticoagulation because of the mechanical aortic valve. The HAS-BLED score was initially developed to predict the risk of potential bleeding in anticoagulated patients affected by atrial fibrillation, however, recent study showed that it was associated with increased risk of major bleeding events after cardiac surgery procedures [6]. In this patient, the HAS-BLED score was 1 and therefore the bleeding risk was estimated relatively low.

In case of hemodynamic collapse consecutive to cardiac cavities compression by hematoma, early detection is mandatory, and POCUS is crucial in this regard for prompt and efficient management [7]. In this patient, POCUS was useful for diagnosis of a compressive hematoma, also to rule out aortic prosthesis dysfunction. While the preliminary echo diagnosis was a compressive hematoma, the presence of foreign body post thoracotomy was raised. When transthoracic echocardiographic findings are suboptimal, transesophageal approach is required [4]. It is noteworthy that in such scenario, most patients are in intensive care, in dorsal supine position, with chest dressing and possibly intubated, making the POCUS via transthoracic approach a challenging exam [7]. Therefore, multimodality imaging including computed tomography is necessary when transthoracic or transesophageal echo findings are suboptimal or non conclusive [5, 7].

## Conclusion

A loculated and compressive pericardial hematoma that occurs after cardiac surgery may lead to hemodynamic collapse. Therefore, close monitoring of hemodynamic parameters is essential during the postoperative period. Additionally, performing POCUS is essential for early recognition and prompt management of this potentially life-threatening condition.

## Data Availability

Non applicable.
